# The Epidemiology and Population-Based Studies of Women with Lower Urinary Tract Symptoms: A Systematic Review

**DOI:** 10.5152/tud.2022.21325

**Published:** 2022-03-01

**Authors:** Ahmet Tahra, Ömer Bayrak, Roger Dmochowski

**Affiliations:** 1 Department of Urology, İstanbul Medeniyet University Faculty of Medicine, İstanbul, Turkey; 2 Department of Urology, University of Gaziantep Faculty of Medicine, Gaziantep, Turkey; 3 Department of Urologic Surgery, Vanderbilt University Medical Center, Tennessee, US

**Keywords:** Epidemiology, female, lower urinary tract symptom, population-based studies

## Abstract

In this systematic review, we focused on epidemiology and population-based studies to identify recent real-world data of women with lower urinary tract symptoms. The PubMed, Scopus, and Cochrane databases were used for the literature search using the following keywords: epidemiology, population-based studies, women, female, lower urinary tract symptoms, and urinary incontinence. A total of 20 articles in the English language were found to be eligible for this review. The prevalence of LUTS in women was 11.8%-88.5%. The prevalence of storage symptoms was 23.6%-79%, voiding symptoms was 1.8%-51%, and post-micturition symptoms was 0.3%-46%. The prevalence of voiding and storage symptoms was 8.3%-26.6% and the prevalence of combined voiding, storage, and post-micturition symptoms was 6.6%-19.2%. Any incontinence was observed in 5.8%-45.8% of women. The majority of patients suffered from stress urinary incontinence with 1.9%-31.8%. The prevalence of urgency urinary incontinence and mixed-type urinary incontinence was 0.7%-24.4% and 2.1%-12%, respectively. Increased age, marital and work status, comorbidities, alcohol consumption, higher parity, vaginal delivery, instrumental delivery, prolonged labor, laceration, and post-menopausal status were found to be risk factors for lower urinary tract symptoms. The prevalence of lower urinary tract symptoms in women is increasing, especially with age. Since the worldwide prevalence of lower urinary tract symptoms remains unknown, multi-continental studies, especially in the developing world, with less heterogeneity and more standardized definitions, are needed to better evaluate real-world data in women with lower urinary tract symptoms.

## Main Points

- Lower urinary tract symptoms (LUTS) are quite common in women.
- The definition of LUTS, the selection and exclusion criteria of patients, and heterogeneity are the most common difficulties for evaluating the real impact of LUTS.
- Further studies evaluating LUTS in women are needed, especially in developing and least-developed countries.

## Introduction

Lower urinary tract symptoms (LUTS) in women are classified as storage symptoms, voiding symptoms, and symptoms after micturition. The most common storage symptoms are urinary frequency, urgency, incontinence, and nocturia, and the most common voiding symptoms are hesitancy, weak stream, splitting or spraying, intermittency, and straining. A feeling of incomplete emptying and post-micturition dribble are the most common post-micturition symptoms.^1,2^

These symptoms affect patients’ quality of life (QoL), and nearly a million women suffer from LUTS worldwide.^3^ In a multicenter study, the prevalence of “sometimes” LUTS in women was 76.3% and “often” LUTS in women was 52.5% based on the usage of International Continence Society (ICS) definitions.^4^ Similarly, a recent internet-based survey showed that the prevalence of LUTS in women over 40 years was 66%.^5^

The definition of LUTS, the selection and exclusion criteria of patients, and heterogeneity are the most common difficulties for evaluating the real impact of LUTS. In this systematic review, we focused on epidemiology and population-based studies to identify recent real-world data for women with LUTS. 

## Materials and Methods

This study complied with the Preferred Reporting Items for Systematic Reviews and Meta-analyses statement for conducting systematic reviews and we also followed the “Synthesis Without Meta-analysis (SWiM) protocol” for this review.

### Eligibility Criteria

According to the PICo (Population,Interest and Context) tool, the population should include women over 18 years of age. The prevalence of LUTS in women is the main interest. The context of this review was the whole world. All population-based studies about LUTS were reviewed. Studies that questioned the prevalence of women (over 18 years old) with LUTS were included in this review. Studies that addressed patients under 18 years old, including only male patients, questioned the treatment effect of LUTS, and focused only on urinary incontinence (UI) were excluded from the final review.

### Information Sources and Search Strategy

Systematic research was conducted between January and August 2021. The PubMed, Scopus, and Cochrane databases were used for the literature search using the following keywords: epidemiology, population-based studies, women, female, lower urinary tract symptoms, and UI. All filters were turned off while searching PubMed. Studies were reviewed and data were extracted by 2 authors (A.T. and Ö.B) independently. We did not use any automated tools. 

### Data Items and Study Risk of Bias Assessment

Primarily, the prevalence of LUTS in women was reviewed. The combined and separate prevalence of voiding, storage, and post-micturition symptoms was also reviewed. The studies that mentioned the prevalence of LUTS and incontinence were also reviewed. Study risk of bias and quality assessment were done using an “assessing risk of bias in prevalence studies” tool.^6^

## Synthesis Methods

According to SWiM guidelines, summarizing effect estimates methods were used. Structured tables were used to evaluate the prevalence of LUTS, the combined and separate prevalence of voiding, storage and post-micturition symptoms, and incontinence.

## Results

### Search Results

**Figure 1.**
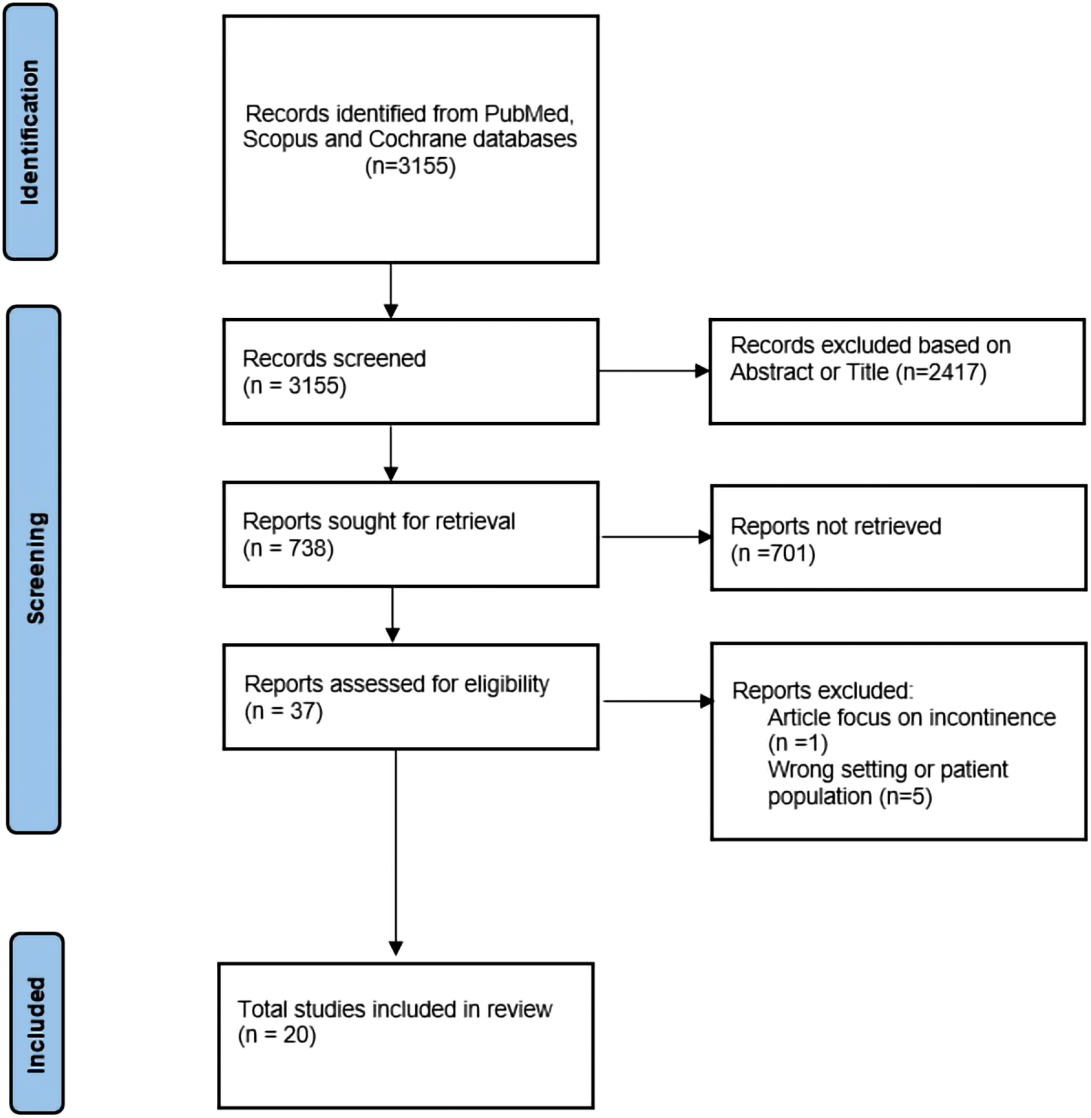
The flowchart of the search strategy for this review.

A total of 3155 articles were selected. Both observational and population-based studies were included. Abstracts, posters, editorials, and review articles were excluded. After which 738 articles with full text were reviewed and included in the final analysis. A total of 20 articles in the English language were found to be eligible for this review (Figure 1). 

### Study Risk of Bias and Quality Results

The assessment of quality and bias are shown in Table 1. According to this tool, all included studies had a low risk of bias. 

### Prevalence of Lower Urinary Tract Symptoms in Women

The prevalence of LUTS in women was 11.8%-88.5% based on a variety of definitions (Table 2).^4,7-25^ Based on definitions, type of questionnaire, and heterogeneity of the population, the prevalence of LUTS in women differed between studies. Most of the studies used the ICS definitions, but cut-off scores for the International Prostate Symptom Score (IPSS) and American Urological Association symptom index were also used. Different validated questionnaires were used, and some authors also used the “Designed Questionnaire” for the studies.^16,18,25^ The minimum age of the participants was 18 years. Of the 20 studies, 15 were from a single country, 3 were from 3 countries, and 2 of them were from more than 3 countries across 4 continents. 

### The Prevalence of Voiding, Storage, and Post-Micturition Symptoms

The prevalence of voiding and storage symptoms is given in Table 3. Twelve studies mentioned the prevalence of voiding, storage, and post-micturition symptoms.^8,10,11,13,15,17,18,21-25^ The prevalence of storage symptoms was 23.6%-79%, voiding symptoms was 1.8%-51%, and post-micturition symptoms was 0.3%-46%. 

### The Prevalence of Combined Voiding, Storage, and Post-Micturition Symptoms

The prevalence of combined voiding, storage, and post-micturition symptoms are given in Table 4.^8,10,13,15,17,21,22^ Only 7 studies mentioned combined voiding and storage symptoms. The prevalence of voiding and storage symptoms was 8.3%-26.6% and the combined voiding, storage, and post-micturition symptoms were 6.6%-19.2%.

### The Prevalence of Urinary Incontinence

Urinary incontinence is reviewed in Table 5. Fifteen studies mentioned UI with variations in definition.^4,9-11,13,14,17-25^ The prevalence of any incontinence was 5.8%-45.8%. The majority of patients suffered from stress urinary incontinence (SUI) with 1.9%-31.8%. The prevalence of urgency urinary incontinence (UUI) and mixed-type urinary incontinence (MUI) was 0.7%-24.4% and 2.1%-12%, respectively. 

### Risk Factors for Lower Urinary Tract Symptoms

Risk factors for LUTS in women are shown in Table 6. Increased age was found to be a definitive risk factor for LUTS.^4,7-12,15,17,19,23-25^ Marital and work status, comorbidities, alcohol consumption, body mass index (BMI), smoking status, neurological disease, educational status, higher parity, vaginal delivery, instrumental delivery, prolonged labor, laceration, post-menopausal status, and physical activity were also studied risk factors for LUTS.^7,8,15,23,24^

### The Most Bothersome Symptoms and Healthcare-Seeking Behaviors

The most bothersome or reported symptoms and healthcare-seeking behaviors are shown in Table 7. The coping methods used for bladder symptoms and the reasons for not seeking help were the symptoms were also summarized.

## Discussion

We designed a systematic review of epidemiology and population studies in women with LUTS. Mostly, the authors used the ICS definitions to evaluate LUTS. We found 20 eligible studies from the data of 20 countries across 4 continents. In this review, we found that 11.8%-88.5% of women experienced LUTS. The most common symptoms of LUTS were storage symptoms, with 23.6%-79% of women in 12 studies. 

In this review, 15 of 20 selected articles were evaluated for UI. Any incontinence was observed in 5.8%-45.8% of women. The most common type of incontinence was SUI with 1.9%-31.8%. Similar to our review, a recent population-based study that investigated the prevalence of incontinence showed that the prevalence of any incontinence was 26.4% in women, but MUI was the most prevalent with 12.6%, followed by SUI and UUI.^26^ In a recent systematic review, the prevalence of UI was found to be 25.7%, with SUI being 12.6%, UUI being 5.3%, and MUI being 9.1%.^27^ Analysis of a health screening project which evaluated the incidence and remission of UI in women showed that baseline prevalence of UI was 32%, and after 6.5 years, it had increased to 43%. The annual incidence of UI was found to be 3.6%, which increased with age.^28^ Heidler et al^29^ also assessed LUTS in women and showed that the prevalence of LUTS increased from 35.9% to 47.1% in 6.5 years and that the annual incidence of LUTS was 5.3%.

Age was found to be the most common risk factor for LUTS. In this review, we showed that the likelihood of experiencing LUTS increases with age. In contrast to our review, van Breda HM et al^30^ showed that the prevalence of LUTS was 94.3%, even in healthy nulligravida women of 18-30 years old. Urinary incontinence may occur even in the early years of life; 41% of female athletes (median age of 22) experienced at least 1 SUI. Nearly 25% of women under 40 also experienced UI during physical activities.^31^ In line with our results, Abufaraj et al^32^ showed that higher age was associated with a higher prevalence of any UI. The 5-year incidence of LUTS was found to be 13.9% in women. The authors showed that the incidence of LUTS increased with age.^33^

Alcohol consumption was the main changeable risk factor for LUTS, while physical activity was solely not a risk factor for LUTS. A study that summarized the literature on LUTS in women (mainly focused on incontinence and overactive bladder) showed that age, smoking, pregnancy, asthma, obesity, dementia, vaginal delivery, constipation, diuretics, and drugs were risk factors for UI.^34^ Parity, fetal birth weight, and episiotomy were also found to be risk factors for women with LUTS.^35^ In a study from Turkey, recurrent urinary tract infection, chronic illness, chronic constipation, and higher BMI were found to be the most common risk factors for women with LUTS.^36^ Urinary symptoms were also found to be more common in women with pelvic organ prolapse.^37^ Ethnicity was also another factor for UI. A cross-sectional survey of American Indian women from 1 tribe showed that the study group had similar rates of mixed incontinence, but stress and urge incontinence rates were lower than previous reports.^38^

All symptoms of LUT were found to be bothersome, but the most visiting issue for women with LUTS was IPSS severity. Loss of bladder control had a higher impact on QoL. Limited fluid intake and wearing absorbent products are the most common coping methods for living with LUTS. Being seen as part of aging and embarrassment from the symptoms are the most common reasons for unwillingness to seek healthcare for the loss of bladder control. Losada et al^31^ showed that over 88% of women experienced a negative impact on their concentration, physical activity and confidence, as well as an inability to finish their work, due to their urinary symptoms. Sumarsono et al^26^ also found that mental and physical QoL were lower in women with UI compared to women without UI. They mentioned that patients with UI had higher anxiety and depression scores. 43.9% of women with UI visited healthcare professionals for their urinary symptoms. A study by Waetjen et al^39^ found that more frequent, more bothersome, and worsening urinary symptoms with a longer symptom duration were the most common factors for seeking treatment in women with UI. Long-term voluntary drug-taking and talking to other individuals about incontinence were also other predictive factors of help-seeking.^40^

Although sexual dysfunction was not a topic for this review, UI has a significant impact on sexual function. Lim et al^41^ showed that patients with SUI and their partners had problems with sexual functioning. They also showed that patients with SUI had a lower QoL score compared to patients without SUI. Salonia et al^42^ found that 46% of women with UI and LUTS had female sexual dysfunction (FSD). The most common findings in FSD were hypoactive sexual desire, arousal disorder, orgasmic deficiency, and dyspareunia or genital pain.^42^

The main limitation of this study is that it is not a meta-analysis. Our inability to perform meta-analysis and heterogeneity was the most significant issue in producing a robust result. The inclusion of patients from different geographies and different age groups, the evaluation of the entire population including men and women, and most importantly, the lack of use of a standardized definition for LUTS were observed as the main causes of heterogeneity. We did not mainly focus on UI so we did not include studies that evaluated types of UI with specific questionnaires like medical, epidemiologic, and social aspects of aging. The evaluation of only 20 countries may mean that adequate representation is lacking in the results, and the prevalence of LUTS in developing and undeveloped countries is still unknown. 

## Conclusion

The prevalence of LUTS in women is increasing, especially with age. Several factors are affecting the prevalence of LUTS, including the fact that only a small proportion of individuals with LUTS seek healthcare. Therefore, prevention and providing necessary treatments are important for women’s health. Since the worldwide prevalence of LUTS remains unknown, multi-continental studies, especially in the developing world, with less heterogeneity and more standardized definitions, are needed to better evaluate real-world data in women with LUTS.

## Figures and Tables

**Table 1.** Quality Assessment for Prevalence Studies Included

**Author**	Q1	Q2	Q3	Q4	Q5	Q6	Q7	Q8	Q9	Q10	Summary
Boyle et al^7^	L	L	L	H	L	L	L	L	H	L	L
Chapple et al^8^	L	L	L	L	L	L	L	L	H	L	L
Coyne et al^4^	L	L	L	H	L	L	L	L	H	H	L
Herschorn et al^9^	L	L	L	H	L	L	L	L	H	L	L
Irwin et al^10^	L	L	L	H	L	L	L	L	L	H	L
Kogan et al^11^	L	L	H	H	L	L	L	L	H	L	L
Kupelian et al^12^	L	L	L	H	L	L	L	L	H	L	L
Lee et al^13^	L	L	L	H	L	L	L	L	H	L	L
Liao et al^14^	L	L	H	L	L	L	L	L	L	L	L
Liu et al^15^	L	L	L	H	L	L	L	L	H	L	L
Møller et al^16^	L	L	L	L	L	H	L	L	L	L	L
Moreira et al^17^	L	L	L	L	L	L	L	L	H	L	L
Mourad et al^18^	L	L	L	H	L	L	L	L	H	L	L
Osuga et al^19^	L	L	H	L	L	L	H	L	L	L	L
Plata et al^20^	L	L	H	L	L	L	L	L	L	h	L
Przydacz et al^21^	L	L	L	L	L	L	L	L	L	L	L
Soler et al^22^	L	L	L	H	L	L	L	L	L	L	L
Wang et al^23^	L	L	L	L	L	L	L	L	H	L	L
Zhang et al^24^	L	L	L	L	L	L	L	L	L	L	L
Zumrutbas et al^25^	L	L	L	H	L	L	L	L	H	L	L

L, low risk; M, moderate risk; H, high risk.

**Table 2.** The Prevalence of Women with Lower Urinary Tract Symptoms

Author	Year	Study Method	Number of Women participants	Number of any LUTS	Prevalence (%)	Age	Validated Questionnaire	Definition of LUTS	Country
Boyle et al^7^	2003	A population-based, cross-sectional survey	3760	709	18.8	40-79	IPSS	Moderate to severe LUTS (IPSS>7)	Netherlands, France, UK Korea
Chapple et al^8^	2017	Cross-sectional, population-representative, Internet-based	4209	2511	59.7	≥40	IPSS and PPBC	ICS definitions	China, Taiwan and South Korea
Coyne et al^4^	2009	Cross-sectional, population-representative survey	15 861	12102	76.3	≥40	SF-12, PPBC, HADS IPSS, OAB-q SF	ICS definitions	USA, the UK and Sweden
Herschorn et al^9^	2007	Population-based telephone cross-sectional survey	509	294-212	56.8	≥18	CATI	ICS definitions	Canada
Irwin et al^10^	2006	Population-based, cross-sectional telephone survey	12 093	8054	66.6	≥18	CATI Questionnaire, IPSS	ICS definitions	Sweden, Germany, Italy, Canada, and the United Kingdom
Kogan et al^11^	2014	Population-based, cross-sectional survey	1614	1356	84	≥18	CATI	ICS definitions	Russia, Czech Republic, and Turkey
Kupelian et al^12^	2006	Population-based, random sample, epidemiologic survey	3205	596	18.6	30-79	AUA-SI, SF-12	AUA-SI score of 8 or above	United States
Lee et al^13^	2011	Population-based, cross-sectional telephone survey	1112	767	68.9	≥18	CATI Questionnaire, IPSS	ICS definitions	Korea
Liao et al^14^	2009	Cross-sectional, questionnaire survey.	907	590	65	21–59	TNBS, SF-36	ICS definitions	Taiwan
Liu et al^15^	2019	Internet-based, self-administered survey	1063	604	56.8	≥40	IPPS, OAB	ICS definitions	Taiwan
Møller et al^16^	2000	Mail-based questionnaire	2854	794	27.8	≥40	BF-LUTS and Designed Questionnaire	ICS definitions	Denmark
Moreira et al^17^	2013	Cross-sectional, population-based survey	1500	1262	84.1	≥30	OAB-V8 and PPBC item	ICS definitions	Brazil
Mourad et al^18^	2018	Population-based, cross-sectional survey	1853	1631	88	≥18	Designed Questionnaire	ICS definitions	Egypt
Osuga et al^19^	2013	Cross-sectional analyses	1218	144	11.8	≥40	IPSS and ICIQ-SF	IPSS ≥8	Japan
Plata et al^20^	2018	Cross-sectional, population-based	530	493	88.5	≥18	IPSS and ICIQ-OAB	IUGA/ICS definitions	Colombia
Przydacz et al^21^	2020	Population-based, cross-sectional analysis	3393	2465-1817	72.6	≥40	CATI Questionnaire, IPSS, OAB-V8	ICS Definitions	Poland
Soler et al^22^	2017	Computer-assisted telephone survey	2751	2262-1624	82.2	≥40	IPSS and OAB-V8	ICS Definitions	Brazil
Wang et al^23^	2015	Population-based, cross-sectional survey	1472	900	61.1	≥18	ICIQ-FLUTS LF	ICS definitions	China
Zhang et al^24^	2015	Population-based, cross-sectional survey	18 992	10540	55.5	≥20	BF-LUTS and ICIQ-FLUTS	ICS definitions	China
Zumrutbas et al^25^	2014	Cross-sectional, population-based survey	919	672	73.5	≥18	Designed Questionnaire, ICIQ-SF	ICS definitions	Turkey

LUTS, lower urinary tract symptoms; BF-LUTS, Bristol Female Lower Urinary Tract Symptoms questionnaire; ICIQ-FLUTS, Incontinence Questionnaire Female Lower Urinary Tract Symptoms; OAB V8, Overactive Bladder-Validated 8; PPBC, the patient perception of bladder condition; IPSS, The International Prostate Symptom Score; ICIQ-FLUTS LF, International Consultation on Incontinence Questionnaire Female Lower Urinary Tract Symptoms Long Form; ICIQ-OAB, Incontinence Questionnaire Overactive Bladder; CATI, Computer-assisted Telephone Interview; SF-12, Short Form Survey 12; HADS, Hospital Anxiety and Depression Scale; OAB-q SF, Overactive Bladder Questionnaire Short Form; AUA-SI, American Urological Association Symptom Index; TNBS, Taiwan Nurse Bladder Survey.

**Table 3.** The Prevalence of Voiding and Storage Symptoms

Author	Number of Participants with SS	SS Prevalence (%)	Number of Participants with VS	VS Prevalence (%)	Number of Participants with PM	PM Prevalence (%)
Chapple et al^8^	1003	23.8	77	1.8	67	1.6
Irwin et al^10^**	7130	59.2	2443	19.5	1695	14.2
Kogan et al^11^	1227	76	598	37	372	23
Lee et al^13^	717	64.4	289	25.9	155	13.9
Liu et al^15^	251	23.6	25	2.3	27	2.5
Moreira et al^17^	1146	76.4	505	33.7	192	12.8
Mourad et al^18^**	1464	79	946	51	853	46
Przydacz et al^21^	1334	39.3	102	3	21	0.6
Soler et al^22^	1002	36.4	122	4.4	9	0.3
Wang et al^23^**	891	60.5	130	8.8	93	6.3
Zhang et al^24^	10 245	53.9	2444	12.8	-	-
Zumrutbas et al^25^	586	64.1	341	37.8	262	28.7

SS, storage symptoms; VS, voiding symptoms; PM, post-micturition symptoms.

**Definitions based on any symptom with nocturia >1.

**Table 4.** The prevalence of combined voiding, store and post-micturition symptoms

Author	Number V + S	Prevalence of V + S (%)	Number V + PM	Prevalence of V + PM (%)	Number S + PM	Prevalence of S + PM (%)	Number V + S + PM	Prevalence of V + S + PM (%)
Chapple et al^8^	393	9.3	42	1	121	2.9	808	19.2
Irwin et al^10^	1855	14.9	931	7.7	1348	11.3	797	6.6
Lee et al^13^	251	22.5	-	-	-	-	87	7.8
Liu et al^15^	89	8.3	20	1.8	42	3.9	154	14.4
Przydacz et al^21^	492	14.5	19	0.5	109	3.2	391	11.5
Moreira et al^17^	399	26.6	117	7.8	167	11.1	96	6.7
Soler et al^22^	518	18.8	18	0.6	141	5.1	457	16.6

V, voiding symptom; S, storage symptoms; PM, post-micturition symptoms.

**Table 5.** The Prevalence of Urinary Incontinence Based on Several Definitions

Author	Definitions of UI	Incontinence (N)	Prevalence Incontinence (%)	SUI (n)	Prevalence SUI (%)	UUI (n)	Prevalence UUI (%)	MUI (n)	Prevalence MUI (%)	Other UI (n)	Prevalence Other UI (%)
Coyne et al^4^	Any incontinence, previous 4 weeks	-	-	5004	31.8	3869	24.4	-	-	1833	9.8
Herschorn et al^9^	Any incontinence	-	-	132	25.5	48	9.3				
Irwin et al^10^	Any incontinence	1675	13.1	721	6.4	208	1.5	317	2.4	329	2.8
Kogan et al^11^	Any incontinence	363	20	113	7	67	4	99	6	33	2
Lee et al^13^	Any incontinence	316	28.4	230	20.7	40	3.6	46	4.1	-	-
Liao et al^14^	During past 12 months, any incontinence	83	9.1	26	2.8	15	1.6	42	4.6	-	-
Moreira et al^17^	Any incontinence	87	5.8	27	1.9	11	0.7	47	3.1	-	-
Mourad et al^18^	Any incontinence	501	27	112	6	112	6	224	12	56	3
Osuga et al^19^	Voluntary loss of urine at least twice or more a week.	80	6.6	66	5.4	40	3.3	26	2.1	-	-
Plata et al^20^	Past 4 weeks, any incontinence	255	45.8	76	13.7	120	21.5	59	10.6	-	-
Przydacz et al^21^	Any incontinence	1242	36.6	415	12.2	316	9.3	348	10.3	163	4.8
Soler et al^22^	Symptoms occurring less than half the time or more	1255	45.6	562	20.4	409	14.9	-	-	411	15.3
Wang et al^23^	Past 4 weeks, any incontinence	562	38.1	265	18.0	57	3.8	47	3.2	193	13.1
Zhang et al^24^	Past 4 weeks, any incontinence	6052	31.9	3592	18.9	488	2.6	1789	9.4	-	-
Zumrutbas et al^25^	Any incontinence	354	38.7	194	21.2	75	8.2	82	9	24	2.6

SUI, stress urinary incontinence; UUI, urgency urinary incontinence; MUI, mixed urinary incontinence; other UI, any other urinary incontinence (leak for no reasons, nocturnal enuresis, leak during sexual activity), n, number of patients.

**Table 6.** Risk Factors for Lower Urinary Tract Symptoms (LUTS) in Women

Risk factors for LUTS	Not a risk factor for LUTS
Age^4,7-12,15,17,19,23-25^	Smoking status^7^,^15^
Martial and work status^8^	Educational status^8^
Comorbidities*^8,15,23,24^	Higher BMI^15^
Alcohol consumption^23,24^	Neurological disease^15^
Higher BMI^23,24^	Physical activity^7^
Smoking status^23,24^	
Neurological disease^8^	
Educational status^23^	
Higher parity, vaginal delivery^23,24^	
Instrumental delivery, prolonged labor, laceration, and post-menopausal status^23,24^	

*Comorbidities: diabetes mellitus, heart disease, hypertension, and hyperlipidemia.

LUTS, lower urinary tract symptoms; BMI, body mass index.

**Table 7.** The Most Bothersome Symptoms and Healthcare-Seeking Behaviors

The most bothersome or reported symptoms for LUTS:^4,8,10,11,13,15,17,21-24^	Storage symptoms Nocturia Frequency Urgency Stress urinary incontinence Urge urinary incontinence Mixed urinary incontinence Voiding, storage, and post-micturition symptoms Leaking for no reason Nocturnal enuresis Post-micturition dribble
The most visiting issue for women with LUTS:^8^	IPSS severity
The symptoms with greater impact on patients’ QoL:^11^	Loss of bladder control Overall bladder symptoms
The coping methods used for bladder symptoms:^11,18,19^	Limiting fluid intake Using wearable products Herbal therapies Drugs
The reasons for not seeking help were:^11^	The symptoms not being severe enough The symptoms being attributed to the natural consequence of aging The symptoms were viewed as shameful The patient could self-treat their symptoms.

LUTS, lower urinary tract symptoms; QoL, quality of life; IPSS, The International Prostate Symptom Score.
